# Ciprofloxacin-Induced Erythema Multiforme Major Unmasking Systemic Lupus Erythematosus

**DOI:** 10.7759/cureus.85483

**Published:** 2025-06-06

**Authors:** Paola A Alicea, Ricardo A Muñoz, Vanessa Sepúlveda

**Affiliations:** 1 Internal Medicine, University of Puerto Rico, Medical Sciences Campus, San Juan, PRI

**Keywords:** chronic autoimmune disease, ciprofloxacin skin reaction, drug hypersensitivity reaction, erythema multiforme major, lupus flare, systemic lupus erythematosus

## Abstract

Systemic lupus erythematosus (SLE) is an autoimmune disease with diverse clinical manifestations characterized by periods of remission and relapse. This report describes the case of a 24-year-old woman with a history of hypothyroidism who developed erythema multiforme major (EMM) following exposure to ciprofloxacin, an antibiotic in the fluoroquinolone class. Subsequent rheumatologic evaluation confirmed a diagnosis of underlying SLE, supported by positive serologic markers and clinical criteria. This case highlights ciprofloxacin-induced EMM as a trigger for unmasking SLE, diagnosed via the 2019 European League Against Rheumatism/American College of Rheumatology (EULAR/ACR) criteria, and underscores the need for rheumatologic evaluation in drug-induced hypersensitivity reactions. Early recognition and intervention are crucial to managing SLE and preventing long-term complications.

## Introduction

Systemic lupus erythematosus (SLE) is a complex and chronic autoimmune disorder in which the body's immune system erroneously attacks its own tissues. Although the precise cause of SLE is not yet fully understood, it is thought to result from a combination of genetic, environmental, and hormonal influences. This condition can affect multiple organs throughout the body, including the skin, joints, kidneys, heart, and lungs, leading to a diverse array of symptoms. Common manifestations include joint pain, skin rashes, fatigue, and fever, while more severe cases may result in complications such as kidney damage or cardiovascular issues [1]. Given its unpredictable nature and varied symptom presentation, SLE often necessitates a multidisciplinary approach to management and treatment.

A "lupus flare" refers to a period of acute exacerbation in individuals diagnosed with SLE [2]. Various factors can trigger this condition, including stress, infections, exposure to sunlight, both physical and emotional trauma, and certain medications. Notably, some antibiotics can induce allergic reactions and immune-mediated hypersensitivity reactions that may overlap with symptoms of a lupus flare, which might be a challenging clinical scenario for physicians. Ciprofloxacin, an antibiotic from the fluoroquinolone class, is frequently used to treat bacterial infections, such as urinary tract infections and pneumonia [3]. It has a range of potential adverse effects, including dermatologic manifestations like erythema multiforme (EM). Literature has documented cases where ciprofloxacin has caused drug-induced lupus (DIL) or mimicked an acute flare of SLE symptoms in patients whose disease was previously well-controlled [4-8]. While rare (<5% of drug-induced lupus cases per literature), ciprofloxacin has been linked to lupus-like flares, particularly in genetically predisposed patients [2,5]. This report details the case of a young female patient with undiagnosed SLE, which was discovered soon after her exposure to ciprofloxacin.

## Case presentation

**Figure 1:**
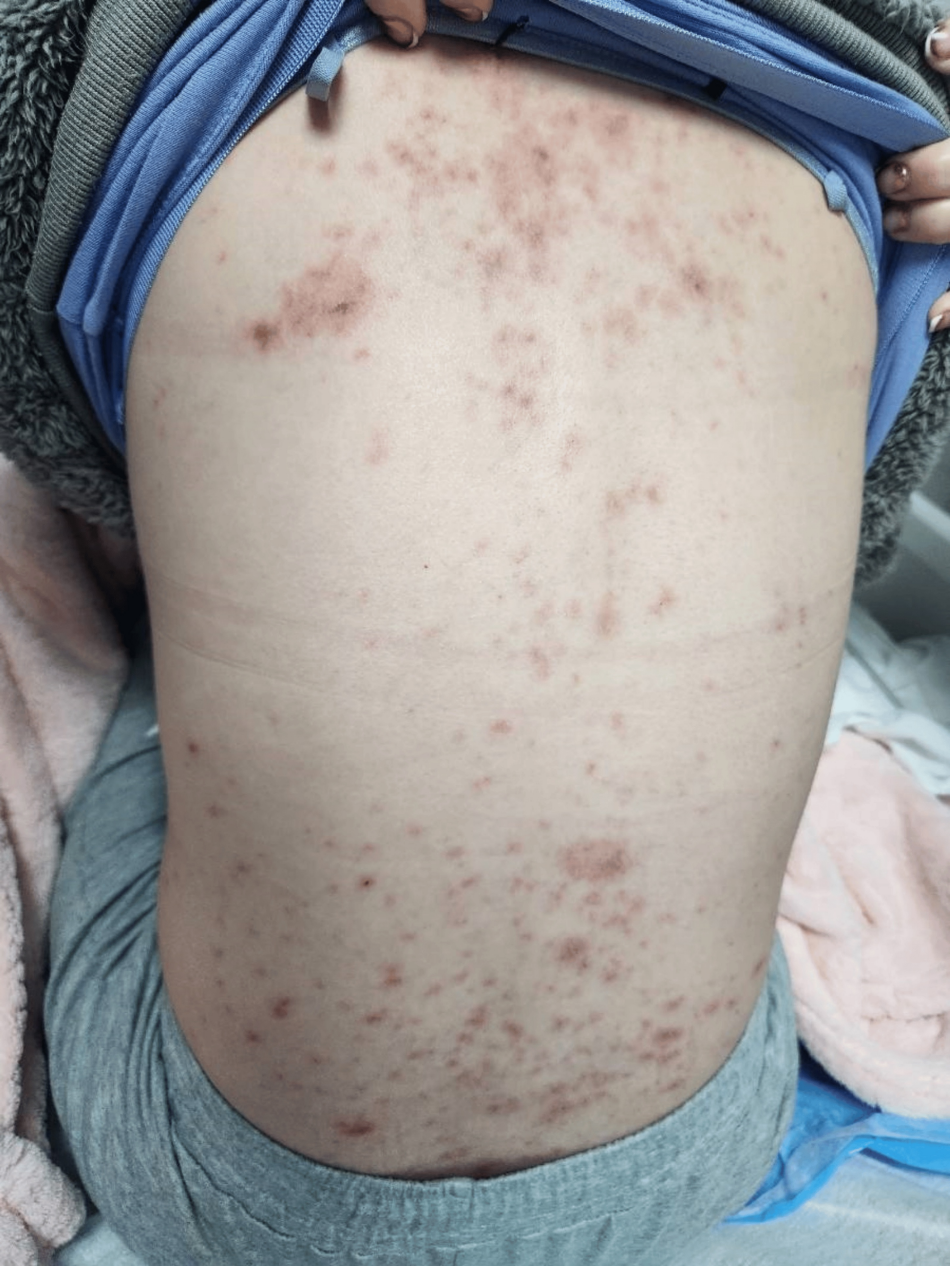
Multiple scattered erythematous targetoid-like papules and plaques located on the back

**Figure 2:**
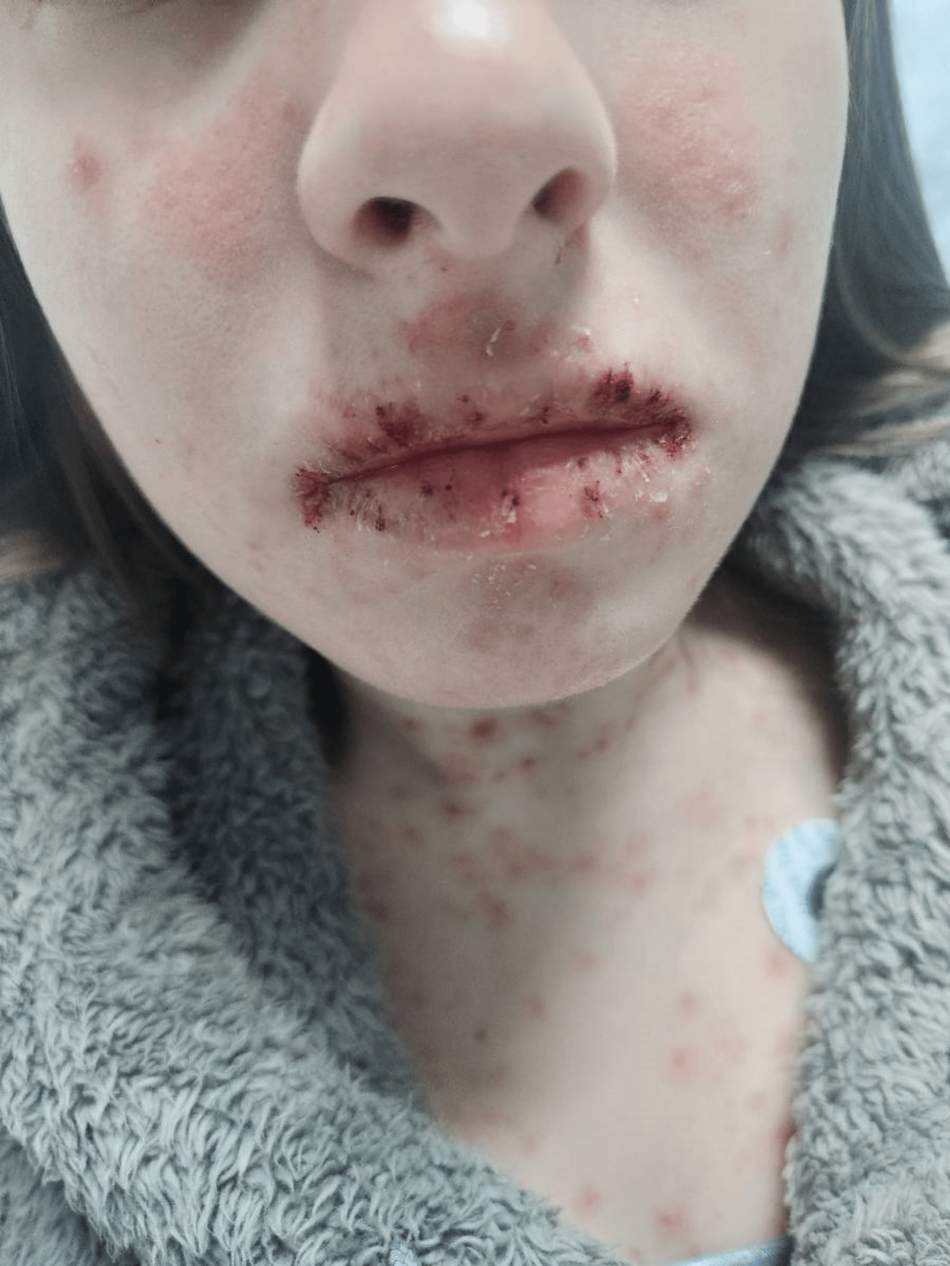
A distinctive malar rash, characterized by a red, butterfly-shaped pattern across the cheeks and nose, and lesions on the skin in both the mouth and neck, are prominently visible.

A 24-year-old woman with a medical history of hypothyroidism presented to the emergency department with a diffuse, burning, and itchy skin rash accompanied by mucosal involvement. The rash began on her abdomen five days after completing a second course of oral ciprofloxacin for a urinary tract infection, spreading centrifugally to her face, upper and lower extremities, and genital and oral mucosa. A general physical examination revealed multiple scattered erythematous targetoid-like papules and plaques, some with central erosions, affecting her mouth, neck, ears, chest, back, arms, legs, palms, and soles (Figure 1). A prominent malar rash, which spared the nasolabial folds, was visible (Figure 2); this was the first time she had noticed it after antibiotic exposure.

The patient denied any systemic symptoms but reported previous intermittent episodes of fatigue and arthralgia since approximately one year ago, specifically in her bilateral fingers and wrists. Family medical history was significant for the presence of Sjögren’s disease and hypothyroidism in the patient’s mother and SLE in her aunt. Given her personal and family history of autoimmune conditions, a comprehensive rheumatologic workup was ordered. Dermatology service was consulted, diagnosing EM major (EMM) as the most probable diagnosis. Topical corticosteroids were applied to the affected areas, and the patient was transferred to the medical intensive care unit (ICU) for close monitoring due to potential progression to Stevens-Johnson syndrome (SJS). 

Upon admission, basic laboratory tests indicated anemia and elevated erythrocyte sedimentation rate (ESR), but no proteinuria, electrolyte disturbances, or other hematologic abnormalities were present (Tables 1-3). Additional results showed positive anti-nuclear antibodies (1:320), low C3 and C4 complement levels, and increased anti-double-stranded DNA (dsDNA) levels (1:80) (Table 4).

**Table 1:** Complete blood count (CBC) shows the presence of anemia but no evidence of leukopenia or thrombocytopenia

Parameters	Patient Values	Reference Ranges
WBC	9.56 Thou/uL	3.98-10.04 Thou/uL
Hemoglobin	10.9 g/dL	13.7-17.5 g/dL
Hematocrit	32.7%	40.1-51.0%
Platlet Count	289 Thou/uL	163-369 Thou/uL

**Table 2:** The basic metabolic panel indicates that renal function is stable and there are no abnormalities in electrolyte levels

Parameters	Patient Values	Reference Ranges
Sodium	139 mmol/L	136-145 mmol/L
Potassium	4.3 mmol/L	3.5-5.1 mmol/L
Chloride	105 mmol/L	98-107 mmol/L
Carbon Dioxide	23.7 mmol/L	22.0-29.0 mmol/L
Blood urea nitrogen	7.5 mg/dL	6.0-20.0 mg/dL
Creatinine	0.58 mg/dL	0.51-0.95 mg/dL
Glucose	87 mg/dL	70-100 mg/dL
Calcium	9.5 mg/dL	8.6-10.0 mg/dL

**Table 3:** Urinalysis shows no signs of proteinuria, hematuria, or urinary casts

Parameters	Patient Values	Reference Ranges
Urine Appearance	Clear	Clear
Urine pH	7.0	4.5-8.0
Urine Protein	Negative	Negative
Urine Blood	Negative	Negative

**Table 4:** Laboratory results indicate an elevated ESR, positive ANA, and anti-dsDNA antibodies, and low C3 and C4 complement levels ESR: erythrocyte sedimentation rate; CRP: C-reactive protein; ANA: antinuclear antibodies; dsDNA: double-stranded DNA; Abs: antibodies; dRVVT: diluted Russels Viper Venom Test; Anti-phospholipid antibodies: anti-cardiolipin and antiB2-glycoprotein IgG, IgM, IgA; RPR: rapid plasma reagin; HIV: human immunodeficiency viruses; Ag/Ab: antigen/antibody; EBV VCA: Epstein-Barr virus viral capsid antigen; Hepatitis Panel: hepatitis A IgM antibody, hepatitis Bs antigen, hepatiris Bs antibody, hepatitis B core Ab, hepatitis C antibody

Inflammatory and Serologic Markers	Patient Values	Reference Ranges
ESR	40 mm/Hr	0-20 mm/Hr
CRP	3 mg/L	0.3-5.0 mg/L
ANA	1:320 Dils	< 1:80 Dils
Anti-dsDNA Abs	1:80 Dils	< 1:10 Dils
Complement C3	57 mg/dL	90-180 mg/dL
Complement C4	9 mg/dL	10-40 mg/dL
Anti-Smith Abs	< 0.2 AI	0.0-1.0 AI
Anti-histone Abs	< 20 U/mL	< 20 U/mL
Lupus anticoagulant	0.808 dRVVT ratio	0.000-1.199 dRVVT ratio
Cardiolipin IgG	< 1.6 U/mL	0.0-20.0 U/mL
Cardiolipin IgM	0.2 U/mL	0.0-20.0 U/mL
Cardiolipin IgA	< 0.5 U/mL	0.0-19.9 U/mL
B2-glycoprotein IgG	< 1.4 U/mL	0.0-20.0 U/mL
B2-glycoprotein IgM	0.2 U/mL	0.0-20.0 U/mL
B2-glycoprotein IgA	< 0.6 U/mL	0.0-20.0 U/mL
RPR	Non-reactive	Non-reactive
HIV Ag/Ab	Non-reactive	Non-reactive
EBV Capsid Ag IgG	13.20 U/mL	< 18.0 U/mL
Hepatitis Panel	Non-reactive	Non-reactive

Three days later, the patient was transferred to the medicine ward following a significant improvement in her skin lesions and the absence of new ones. Dermatology ruled out SJS and confirmed EMM through a skin biopsy, which revealed focal vacuolar interface dermatitis with marked lymphocytic infiltration. Infectious etiologies, including HIV, hepatitis, mononucleosis, COVID-19, mycoplasma, and syphilis, were excluded. Given the suspicion of active disease in a patient without a prior formal diagnosis of SLE, a consultation with rheumatology was requested, underscoring the collaborative approach among various medical specialties. The patient met the 2019 European League Against Rheumatism/American College of Rheumatology (EULAR/ACR) classification criteria for SLE by fulfilling an obligatory entry criterion (ANA > 1:80), along with two clinical criteria, earning 6 points each for acute cutaneous lupus and musculoskeletal involvement. Additionally, she exhibited low levels of C3 and C4 complement proteins, contributing 4 points. This results in a total additive score of 16, which exceeds the required threshold of 10 points. 

Following the exclusion of SJS, the patient's rash improved, eliminating the need for further hospitalization. She was discharged without complications. The primary healthcare team arranged follow-up appointments with rheumatology and dermatology, including reassessing the rheumatological workup after improving inflammation. She began treatment for SLE; prompt rheumatologic follow-up confirmed SLE stability on hydroxychloroquine and prednisone, with no recurrent flares noted upon evaluation at three months.

## Discussion

This case involved a young woman with a notable personal and family history of autoimmune conditions who was previously unaware of the potential for SLE prior to her exposure to ciprofloxacin. Despite experiencing symptoms such as arthralgia and fatigue, she initially attributed these manifestations to her hypothyroidism. This diversion is understandable, as both conditions can present nonspecific symptoms, including joint pain, fatigue, and various skin manifestations [1]. 

Significantly, the onset of her malar rash occurred following the initiation of ciprofloxacin treatment. While it is uncommon for lupus flare-ups to be directly linked to ciprofloxacin, a few documented instances of ciprofloxacin-induced skin reactions mimic the appearance of a lupus flare [2,5]. This context may clarify the laboratory finding of active disease in this patient, characterized by hypocomplementemia (with reduced C3 and C4 levels) and an elevated concentration of anti-dsDNA antibodies in the serum (1:80). 

One of the side effects of ciprofloxacin is EM, which is an acute, self-limiting mucocutaneous hypersensitivity syndrome [6,9]. It has a varied etiology, often recurs, features unusual clinical presentations, and its exact cause is uncertain. In addition to the characteristic rash, individuals may experience fever, joint pain, and general malaise, symptoms similar to those found in SLE. While this can be confusing, it is important to note the timeline of symptoms; the patient had already exhibited arthralgia and fatigue months prior to exposure to ciprofloxacin.

In the case of ciprofloxacin-induced EMM, it is thought to be a result of a delayed-type hypersensitivity reaction, likely triggered by the immune system's response to ciprofloxacin or its metabolites [9]. These reactions are primarily facilitated by T cells, which are critical for coordinating immune responses. Ciprofloxacin may promote T-cell-mediated hypersensitivity via haptenization, exacerbating underlying immune dysregulation in predisposed SLE patients, as seen in this case. There have been various case reports documenting ciprofloxacin-induced EMM in patients with SLE [6,9]. Additionally, this medication may lead to other serious dermatologic reactions, including SJS, toxic epidermal necrolysis (TEN), hypersensitivity, anaphylaxis, erythema nodosum, and photosensitivity, many of which are also associated with SLE [4].

In contrast, DIL is considerably less likely in this clinical context. This autoimmune disorder is characterized by the emergence of lupus-like symptoms, which frequently include fever, musculoskeletal manifestations, and serositis [8]. These symptoms develop after continuous exposure to a drug for more than one month and typically resolve upon discontinuation of the offending medication. DIL usually accompanies serological findings such as a positive ANA test and anti-histone antibodies [8]. Unlike SLE, antibodies to dsDNA are uncommon in this condition. However, this patient tested positive for antibodies to dsDNA while having negative anti-histone antibodies.

## Conclusions

This case highlights how autoimmune conditions, such as SLE, can remain asymptomatic or undiagnosed until provoked by a triggering factor, such as the administration of antibiotics. These triggers can unmask symptoms or initiate a series of effects that make underlying issues more apparent. Therefore, a comprehensive evaluation is essential. It also emphasizes the critical importance of differential diagnosis within the medical field. Even in the absence of symptoms, regular monitoring and aggressive treatment are vital because SLE can rapidly cause organ damage.
